# The aquaglyceroporin AQP9 contributes to the sex-specific effects of *in utero* arsenic exposure on placental gene expression

**DOI:** 10.1186/s12940-017-0267-8

**Published:** 2017-06-14

**Authors:** Emily F. Winterbottom, Devin C. Koestler, Dennis Liang Fei, Eric Wika, Anthony J. Capobianco, Carmen J. Marsit, Margaret R. Karagas, David J. Robbins

**Affiliations:** 1 Molecular Oncology Program, DeWitt Daughtry Family Department of Surgery, University of Miami Miller School of Medicine, Miami, FL 33136 USA; 2 Department of Biostatistics, University of Kansas Medical Center, Kansas City, KS 66160 USA; 3 Department of Pharmacology and Toxicology, Program in Experimental and Molecular Medicine, Geisel School of Medicine at Dartmouth, Hanover, NH 03755 USA; 4 Current address: Weill Cornell Medicine, New York, NY 10065 USA; 5 Sylvester Comprehensive Cancer Center, University of Miami Miller School of Medicine, Miami, FL 33136 USA; 6 Department of Environmental Health, Rollins School of Public Health at Emory University, Atlanta, GA 30322 USA; 7 Department of Epidemiology, Geisel School of Medicine at Dartmouth, Hanover, NH 03755 USA; 8 Department of Biochemistry and Molecular Biology, University of Miami Miller School of Medicine, Miami, FL 33136 USA

**Keywords:** Arsenic, AQP9, *in utero*, Fetal placenta

## Abstract

**Background:**

Sex-specific factors play a major role in human health and disease, including responses to environmental stresses such as toxicant exposure. Increasing evidence suggests that such sex differences also exist during fetal development. In a previous report using the resources of the New Hampshire Birth Cohort Study (NHBCS), we found that low-to-moderate i*n utero* exposure to arsenic, a highly toxic and widespread pollutant, was associated with altered expression of several key developmental genes in the fetal portion of the placenta. These associations were sex-dependent, suggesting that *in utero* arsenic exposure differentially impacts male and female fetuses. In the present study, we investigated the molecular basis for these sex-specific responses to arsenic.

**Methods:**

Using NanoString technology, we further analyzed the fetal placenta samples from the NHBCS for the expression of genes encoding arsenic transporters and metabolic enzymes. Multivariable linear regression analysis was used to examine their relationship with arsenic exposure and with key developmental genes, after stratification by fetal sex.

**Results:**

We found that maternal arsenic exposure was strongly associated with expression of the *AQP9* gene, encoding an aquaglyceroporin transporter, in female but not male fetal placenta. Moreover, *AQP9* expression associated with that of a subset of female-specific arsenic-responsive genes.

**Conclusions:**

Our results suggest that *AQP9* is upregulated in response to arsenic exposure in female, but not male, fetal placenta. Based on these results and prior studies, increased *AQP9* expression may lead to increased arsenic transport in the female fetal placenta, which in turn may alter the expression patterns of key developmental genes that we have previously shown to be associated with arsenic exposure. Thus, this study suggests that AQP9 may play a role in the sex-specific effects of *in utero* arsenic exposure.

**Electronic supplementary material:**

The online version of this article (doi:10.1186/s12940-017-0267-8) contains supplementary material, which is available to authorized users.

## Background

Over the last 20–30 years, the clinical and research communities have become increasingly aware of the importance of accounting for sex differences in both the etiology and treatment of disease. In particular, studies have demonstrated that the effects of drugs and other substances, including environmental toxicants, can differ extensively between men and women [1, 2]. It is now evident that these sexually dimorphic responses exist not only in children and adults, but also in the developing fetus *in utero*, from early stages of development [3–5]. The *in utero* environment, and how the fetus responds to it, are suggested to be key determinants of lifelong health (reviewed in [6]). Thus, there is a crucial need for an improved understanding of the molecular mechanisms underlying sexually dimorphic responses *in utero,* in order to effectively prevent, diagnose and treat the effects of prenatal toxicant exposure.

Arsenic is a ubiquitous natural and anthropomorphic toxicant found in the earth’s crust, food, and water [7]. In some regions of the world, for example Bangladesh, Chile, and Taiwan, populations are exposed to levels of arsenic reaching thousands of micrograms per liter [8]. In addition to having multiple adverse effects on adult health, including skin lesions, metabolic and cardiovascular diseases, and various cancers [9], there is evidence that *in utero* exposure to high levels of arsenic can affect the developing fetus, potentially contributing to increased rates of spontaneous abortion or neonatal death [10, 11], reduced birth weight [12–14], infant infections [15], and chronic disease in later life [16]. Importantly, millions of people worldwide are exposed to arsenic at lower levels than those witnessed in the above populations, but which are close to or exceed the US Environmental Protection Agency Maximum Contaminant Level (EPA MCL) of 10 μg/L [8]. The consequences of this common low-level exposure are poorly understood, but increasing evidence from our laboratories and others suggests that such exposure also impacts fetal tissue biology and adversely affects infant health [17–23].

Notably, several studies have identified sex differences in the effects of prenatal arsenic exposure: Reductions in birth weight and increased infection risk were significantly associated with *in utero* arsenic exposure only among male infants [14, 15], and different relationships between *in utero* arsenic exposure and cognitive function have been observed in male and female children [24]. However, the molecular mechanisms underlying these sex differences have not yet been characterized.

In two recent reports [25, 26], we investigated the effects of *in utero* exposure to arsenic using data from the New Hampshire Birth Cohort Study, a pregnancy cohort from a US region whose residents experience levels of arsenic in drinking water close to and above the EPA MCL, due to the use of unregulated private wells. We measured the expression of a set of candidate genes in the fetal placenta and examined their associations with both levels of total maternal urinary arsenic (U-As) and infant birth weight. Interestingly, in the latter study [25], stratification of the cohort by fetal sex revealed extensive sexual dimorphism in the placental gene expression changes associated with U-As levels, with a different and substantially greater number of associated candidate genes being identified in females than in males. In the present study, we further analyzed gene expression data from our cohort to test the hypothesis that the sexual dimorphism we observed in the placental response to arsenic is related to sex-specific differences in the expression of arsenic metabolism or transport genes.

## Methods

### Study cohort

The study cohort comprised 133 pregnant women enrolled in the ongoing New Hampshire Birth Cohort Study [27]. Eligibility criteria have been described previously [28]. Briefly, participants were English-speaking, mentally competent women of 18–45 years of age, whose home water supply was from a private well, and who had not changed residence since their last menstrual period. Demographic data, pregnancy history and outcome, and lifestyle factor information were collected from questionnaires, and prenatal and delivery records.

### Sample collection and arsenic measurement

Full details of sample collection and arsenic measurement have been described previously [28]. In summary, maternal spot urine samples were collected at approximately 24–28 weeks of gestation and analyzed for levels of individual arsenic species at the University of Arizona, using high-performance liquid chromatography inductively coupled plasma mass spectrometry (ICP-MS). The detection limit for each arsenic species ranged from 0.10 to 0.15 μg/L. Measurements below the limit of detection were recorded as the median value between 0 μg/L and the detection limit for that arsenic species. The total maternal urinary arsenic concentration, U-As, was calculated as the sum of arsenite (As^III^), arsenate (As^V^), dimethylarsinic acid (DMA^V^) and monomethylarsonic acid (MMA^V^), with arsenobetaine excluded.

### Placenta biopsy and gene profiling

At the time of delivery, biopsies were taken at the base of the umbilical cord insertion, immersed immediately in RNAlater (Life Technologies), and stored at −80 **°**C prior to analysis. RNA extraction and expression profiling were performed in three batches. Placental tissue was homogenized in Tri Reagent (Molecular Research Center) using a motorized homogenizer [26]. RNA was extracted as per the manufacturer’s protocol, and further purified using the RNeasy mini kit (Qiagen). RNA quality was determined using an Agilent Bioanalyzer to determine RIN (RNA integrity number) scores, and RNA extractions were repeated where necessary to ensure all RIN scores were >6. Gene expression analysis was performed on 100 ng of RNA per sample using the NanoString system (NanoString Technologies) at the Oncogenomics Core Facility of the University of Miami. The NanoString codeset was custom-designed for specific genes of interest including 29 key developmental/stemness genes, comprising components and consensus target genes of the HH, NOTCH, and WNT signaling pathways and stem cell-related biomarkers (listed in Additional file 1 and [25]); two arsenic metabolism genes (*AS3MT, GSTM1*); two arsenic transporter genes (*AQP9, SLC39A2*); and five housekeeping genes (*ATCB*, *GAPDH*, *HPRT1*, *RPL19*, and *RPLP0*). Two distinct probes were designed for *GLI1* and *PTCH1* (GLI1/GLI1-2, PTCH1/PTCH1-2). Raw count data was first normalized to the spike-in positive controls to account for assay efficiency, and then normalized to the geometric mean expression value of the five housekeeping genes using nSolver software (NanoString Technologies).

### Statistical analysis

Gene expression data was first batch-adjusted using the COMBAT method [29], as in our previous study [25]. Principal component analysis was then performed to ensure that batch effects were successfully attenuated. Using the batch-adjusted data, a series of multivariable linear regression models were used to examine the association between U-As and expression of each of the candidate developmental genes [25, 26], after stratification by infant sex. We modeled natural log-transformed gene expression as a function of log10-transformed U-As, and adjusted for maternal age, based on previously described linear regression analyses to identify potential confounders [25]. We also tested the effect of adjusting our results for urinary specific gravity, as a measure of urine dilution (Additional file 2). Urinary specific gravity data were available for 122 (91.7%) of the 133 participants, and were obtained using a handheld refractometer with automatic temperature compensation (PAL-10S; ATAGO Co Ltd). For the vast majority of genes, this adjustment did not change the directionality of the coefficient estimate, and the majority of estimates were within one standard error of the corresponding estimate obtained in the unadjusted model (Additional file 2). For these reasons, and to optimize our statistical power, we opted to not include adjustment for specific gravity in our final statistical models. Power analysis indicated adequate statistical power for detecting low-moderate correlations between U-As levels and gene expression (absolute correlation (r) = 0.24; power = 80%) at a significance level of 0.05 and a study sample size of 133 subjects [26]. Similar methods were used to examine the associations of developmental gene expression with *AQP9* expression or with percentages of individual arsenic subtypes, and the associations of arsenic metabolism and transport gene expression with U-As. All analyses were conducted using the R statistical program, version 2.13 (http://cran.r-project.org/).

## Results

Our study cohort comprised 133 mother-child pairs enrolled at prenatal clinics in the state of New Hampshire, USA, as part of the ongoing New Hampshire Birth Cohort Study (NHBCS). Demographic information is provided in Table 1. Briefly, the average age at enrollment was 31.1 years and the average gestational period was 39.5 weeks. The majority of participants were non-smoking during pregnancy. The average body mass index (BMI) pre-pregnancy was 24.9 kg/m^2^ and parity was 1.1. Sixty-five (48.9%) of the infants were male and 68 (51.1%) were female, and the average birth weight was 3.4 kg. The median arsenic concentration in household tap water was 0.36 μg/L (interquartile range [IQR] 0.02 – 3.55), with 16% of participants consuming water containing arsenic at concentrations above the EPA MCL of 10 μg/L. The median concentration of total urinary arsenic (U-As), which includes all arsenic species excluding arsenobetaine, was 4.4 μg/L (IQR 1.8 – 11.9).

**Table 1:** Study cohort demographic information

Characteristic	Mean (SD)	Number (%)	Median (interquartile range)
Number of pregnant women		133	-
Gestational age (wks)	39.5 (1.6)	-	-
Maternal age at enrollment (yrs)	31.1 (4.6)	-	-
Parity	1.1 (1.1)	-	-
Pre-pregnancy BMI (kg/m^2^)	24.9 (4.7)	-	-
Smoking status during pregnancy:			
Never	-	97 (72.9)	-
Former	-	13 (9.8)	-
Current	-	5 (3.8)	-
Unknown	-	18 (13.5)	-
Number of infants	-	133	-
Infant birth weight (kg)	3.4 (0.4)	-	-
Infant sex:			
Male	-	65 (48.9)	-
Female	-	68 (51.1)	-
Household water arsenic (μg/L)	-	-	0.36 (0.02 - 3.55)
Total urinary arsenic (U-As, μg/L)	-	-	4.4 (1.8 - 11.9)

In a previous report [25], we used this study cohort to explore the effects of *in utero* arsenic exposure on the key molecular regulators of human fetal development. We analyzed the mRNA expression of 29 candidate genes, comprising stem cell-related biomarkers and components or consensus target genes of the HH, NOTCH, and WNT pathways ([25] and Additional file 1), in the fetal portion of the placenta. We then used multivariable linear regression analysis to identify those genes whose expression associated with maternal U-As levels. We found that, among these 29 candidate genes, seven genes showed association of their expression with maternal U-As, and interestingly, the majority of these associations were dependent on fetal sex. Specifically, in female fetal placenta, *GLI1, GLI3, HES1, LGR5* and *IGFBP6* were associated with U-As levels, while in males, only *PORCN* was significantly associated with U-As [25].

In the present study, we examined the mRNA expression of a set of four genes with known roles in arsenic transport (*AQP9* and *SLC39A2*) or arsenic metabolism (*AS3MT* and *GSTM1)* in male and female fetal placenta, to start to examine the possibility that differences in arsenic transport or metabolism might account for the sexually dimorphic effects of arsenic on the placental expression of these developmental genes. We observed no significant differences in the average expression levels of any of these four genes between male and female placenta (Additional file 3). However, when we examined their associations with U-As levels, we found a strong positive relationship between the expression of *AQP9* (aquaporin 9) and U-As levels in females, but not in males (Table 2). In a previous study, we reported a positive association between *AQP9* expression and U-As levels in the cohort as a whole [26] (coefficient estimate: 0.25; 95% confidence interval: 0.05–0.45); our new analysis reveals that this association is largely among female placentae (coefficient estimate: 0.41; 95% confidence interval: 0.15–0.67).

**Table 2:** *AQP9* expression in female placenta is positively associated with arsenic exposure

	Females			Males		
Gene	Coefficient estimate	Standard error	*P*-value	Coefficient estimate	Standard error	*P*-value
*AQP9*	0.411	0.133	0.003**	0.028	0.156	0.857
*AS3MT*	−0.115	0.074	0.126	0.058	0.082	0.483
*GSTM1*	0.107	0.435	0.806	−0.279	0.506	0.584
*SLC39A2*	−0.105	0.101	0.300	−0.015	0.089	0.870

Multivariable linear regression analyses were performed, after stratification of the cohort by infant sex, to determine the association between maternal U-As and placental expression of arsenic metabolism or transport genes in male and female infants. The analyses were adjusted for maternal age. **P < 0.01

**Fig. 1:**
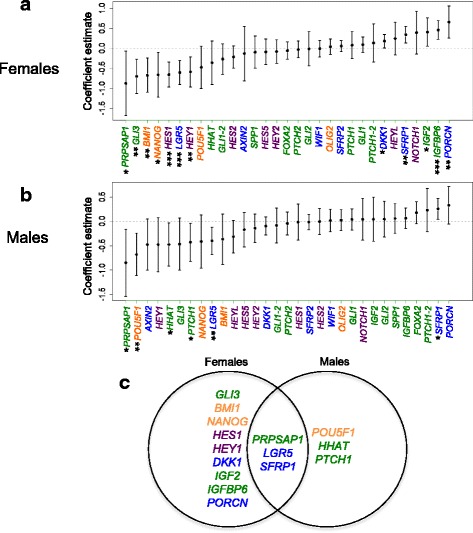
Multiple developmental genes are associated with expression of *AQP9* in fetal placental tissue. Multivariable linear regression analyses were performed to determine the association of placental *AQP9* expression with placental developmental/stemness gene expression, for (**a**) female, and (**b**) male fetal placenta. The analyses were adjusted for maternal age. GLI1/GLI1-2 and PTCH1/PTCH1-2 are sequence-distinct NanoString probes designed to measure the expression of *GLI1* and *PTCH1*. *Dots* depict coefficient estimates and error bars represent 95% CIs. Significant associations are those with 95% CIs not crossing zero (*dotted line*) and are marked by *asterisks* (* *P* < 0.05, **P < 0.01, ***P < 0.001). **c** Venn diagram summarizing *AQP9*-associated genes in males and female placenta. *Green*; HH pathway-related genes, *purple*; NOTCH pathway-related genes, *blue*; WNT pathway-related genes, *orange*; stemness genes

AQP9 is a known transporter of trivalent arsenics [30, 31], besides transporting water, glycerol, urea, and several small, uncharged solutes [32]. We reasoned that increased AQP9-mediated transport, resulting from the observed upregulation of *AQP9* mRNA, might be responsible for some of the effects of arsenic exposure on gene expression in female placenta. Thus, we next tested for sexual dimorphism among developmental candidate genes whose expression relates to that of *AQP9* (Fig. 1). We found that the expression of multiple developmental genes, encoding components of the Wnt, HH and Notch pathways, and stem cell regulators, related to *AQP9* expression in females (Fig. 1a). In males, expression of a smaller but overlapping set of genes related to that of *AQP9* (Fig. 1b-c).

**Fig. 2:**
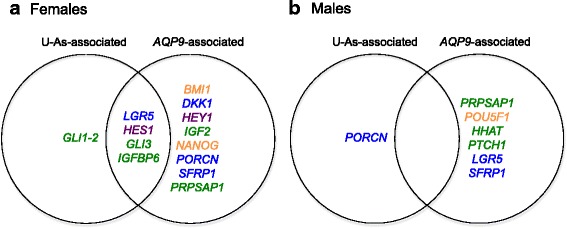
A subset of developmental genes associates with both *AQP9* expression and U-As levels in female fetal placenta. Venn diagrams showing candidate developmental/stemness genes whose expression was significantly associated with U-As levels and/or *APQ9* expression in (**a**) female, or (**b**) male fetal placenta. *Green*; HH pathway-related genes, *purple*; NOTCH pathway-related genes, *blue*; WNT pathway-related genes, *orange*; stemness genes

Comparison of the genes associating with U-As and *AQP9* revealed that four of the five genes whose expression associated with U-As in female placenta—*LGR5*, *HES1, GLI3* and *IGFBP6*—showed similar associations with *AQP9* (Fig. 2a). Moreover, adjustment for *AQP9* expression attenuated the associations of these genes with U-As levels (Additional file 4), suggesting that AQP9 may be an important mediator of arsenic’s effects on these genes. However, the associations of these genes with *AQP9* expression remained significant after adjusting for U-As levels (Additional file 5). A possible interpretation of these results is that, in female placenta, a subset of the effects of arsenic exposure on developmental gene expression may be the result of increased AQP9-mediated transport.

Interestingly, *GLI1* expression was associated with U-As in female placenta, but did not associate with *AQP9* expression (Fig. 2a), and the association between U-As and *GLI1* expression was not affected by adjustment for *AQP9* (Additional file 4). Moreover, in male placenta, *PORCN* expression was negatively associated with U-As levels, but did not associate with *AQP9* expression (Fig. 2b). These observations suggest that arsenic can also affect the placental expression of certain genes independently of AQP9.

Different species of arsenic have been shown to have differing toxicities, and their percentages in urine have been associated differentially with arsenic-related health outcomes (reviewed in [33]). Additionally, the percentages of arsenic metabolites in urine are considered to reflect arsenic methylation capacity. Thus, we next examined whether the expression of our candidate developmental/stemness genes was differentially associated with the percentages of the major arsenic subtypes in urine: inorganic arsenic (%iAs), monomethylarsonic acid (%MMA^V^), and dimethylarsinic acid (%DMA^V^). This analysis revealed that, in female placenta, the expression of several candidate genes was related to the percentages of different arsenic species, in particular %MMA^V^ (Additional file 6). In contrast, the only significant association in male placenta was between *HEYL* and %MMA^V^, consistent with the smaller number of genes found to be associated with total U-As in males. These data indicate that different arsenic species may have differential and sex-specific effects on placental gene expression; and further, that maternal arsenic methylation capacity may impact the effects of arsenic exposure on the fetal placenta.

## Discussion

In a recent analysis of the New Hampshire Birth Cohort, we identified extensive sexual dimorphism in the associations between the placental mRNA expression of key developmental genes and *in utero* arsenic exposure [25]. In the current study, we used gene expression data from our study cohort to consider the potential mechanisms that may underlie this sexual dimorphism. We observed a positive association of maternal U-As levels with expression of the aquaglyceroporin transporter, *AQP9*, in female, but not male, fetal placenta, indicating that *AQP9* may be upregulated in response to arsenic in a female sex-specific manner. This is in keeping with previous studies in animal models, showing upregulation of *AQP9* in response to arsenic [34], as well as sex-specific regulation of *AQP9* expression [35, 36]. These prior studies suggest that estrogen may play a role in facilitating transcription of *AQP9* in liver cells, and suggest it may be useful to examine sex hormone dependent effects in placental tissue as well. Further, we found that the expression of several of our candidate developmental genes associated with *AQP9* expression, and that in females these included a subset of genes that also associate with U-As. A possible explanation for these results is that arsenic acts via regulation of AQP9 to affect the expression of a subset of developmental genes in female fetal placenta, while in both males and females, a distinct subset of genes responds to arsenic independently of AQP9.

AQP9 facilitates cellular uptake of trivalent arsenics [30, 31, 37, 38]. Thus, it is possible that the arsenic-induced upregulation of placental *AQP9* in females increases levels of intracellular trivalent arsenics, increasing their availability to regulate gene expression. While this may be true, we also found that a subset of arsenic-responsive genes, namely *GLI1* and *PORCN*, may be affected by arsenic independently of *AQP9* levels. One possible explanation is that these two subsets of genes are regulated by different arsenic species. Arsenic in drinking water primarily comprises the trivalent and pentavalent inorganic arsenics, arsenite and arsenate, but is metabolized by the liver to methylated arsenic species, such as monomethylarsonic acid (MMA^V^) and dimethylarsinic acid (DMA^V^). Therefore, arsenic in maternal and fetal cord blood contains a mixture of these different species and potentially others [39]. Thus, one possibility is that AQP9 transports a specific trivalent arsenic species into placental cells to affect the expression of one set of genes (“AQP9-dependent genes”), while other arsenic species, e.g., pentavalent species, enter cells independently of AQP9 to regulate a distinct set of genes (“AQP9-independent genes”). However, our analyses relating placental gene expression with percentages of different arsenic species did not lend support to such a model. Indeed, the associations of both AQP9-dependent and -independent genes with %DMA^V^ most closely resembled their associations with total U-As, suggesting that this species might be the primary regulator of both gene subsets. However, due to continuing metabolism, arsenic species percentages in fetal blood differ from those in maternal urine, and so caution should be taken when drawing conclusions regarding species-specific effects from such data.

Notably, a recent analysis of the NHBCS cohort did not reveal significant differences in total placental arsenic concentrations between male and female placentae [40]. This would seem contrary to a model in which AQP9 induces gene expression changes by increasing placental intracellular arsenic levels. One explanation for this outcome is that, unlike urinary arsenic, measurements of placental arsenic do not exclude arsenobetaine (due to the inability to distinguish arsenic species at the relatively low levels found in the placenta). Depending on diet, arsenobetaine can constitute a large proportion of excreted arsenic [41], but is thought to be non-toxic and thus was excluded in our calculations of total urinary arsenic. The inclusion of arsenobetaine may confound the analysis, preventing the identification of sex differences in placental arsenic levels. An alternative possibility is that the observed AQP9-dependent gene expression changes result from increased AQP9-mediated transport of substances other than arsenic. In addition to arsenic, AQP9 is capable of transporting water, glycerol, urea, and other small, uncharged solutes [32]. The arsenic-induced increase in *AQP9* expression could result in altered levels of these substrates, some of which may cause changes in gene expression and cell function. The activity and normal function of placental AQP9 are unknown, although, interestingly, *AQP9* is also upregulated in preeclampsia [42], suggesting it may have a wider connection with intrauterine stress.

Our data indicate that AQP9-mediated transport underlies the female-specific regulation of several key developmental genes in response to arsenic exposure. However, it should be noted that the genes that associate with U-As independently of AQP9 are distinct in males and females—*PORCN* and *GLI1* respectively—indicating that other mechanisms also contribute to sex differences in the effects of *in utero* arsenic exposure.

The candidate genes whose placental expression we have identified as being associated with arsenic exposure encode components and/or targets of the HH, Wnt and Notch signaling pathways, as well as the stem cell regulator POU5F1 (OCT4). The possible implications of the changes we observed in the expression of these genes were discussed in our previous paper [25]. Such changes may interfere with placental development and function, contributing to adverse pregnancy outcomes. For example, alterations in *POU5F1* expression have been associated with gestational trophoblastic disease [43]. Moreover, since arsenic readily crosses the placenta [44], the gene expression changes that we observed in the placenta might also occur in the developing fetus. Heterozygous mutations in several of these genes have been associated with congenital disorders, for example, *GLI3* in polydactyly syndromes [45], and *PORCN* in focal dermal hypoplasia (Goltz syndrome) [46, 47], suggesting that the changes that we observe in relation to arsenic exposure may have important implications for infant and long-term health.

In our previous study, we showed that the expression of *GLI3* and *LGR5* in female fetal placenta was not only negatively associated with U-As levels, but also positively associated with birth weight, identifying these genes as possible mediators of arsenic’s effects on female fetal growth [25]. Interestingly, in our current study both of these genes associated with *AQP9* expression. This suggests that AQP9 may mediate arsenic’s effects on the placental expression of some key regulators of female fetal growth, and thus could play an important role in the effects of arsenic on female infant birth weight and potentially other health outcomes.

## Conclusions

This report identifies AQP9 as a potential mediator of a subset of the sex-specific effects of arsenic exposure on gene expression in the female fetal placenta. Moreover, our findings further support the now abundant evidence in the literature that the fetal placenta exhibits substantial sexual dimorphism, both in its molecular responses to changes in the intrauterine environment, and in how these are translated to functional effects and infant health outcomes (reviewed in [4, 5, 48]). While the mechanisms underlying these sex differences are still being elucidated, it is clear that such differences must be acknowledged and considered in studies of prenatal disease and exposure.

## Additional files

Additional file 1: Developmental/stemness candidate genes. (XLSX 32 kb)

Additional file 2: Associations of arsenic exposure with candidate developmental/stemness and arsenic metabolism/transport genes, with and without adjustment for urinary specific gravity. Multivariable linear regression analyses were performed to determine the association of U-As levels with developmental/stemness gene expression in female (sheet 1) or male (sheet 2) fetal placenta, with or without adjustment for urinary specific gravity as indicated. All analyses were adjusted for maternal age. Genes showing differences in coefficient estimates between models of greater than one standard error are highlighted in yellow. (XLSX 72 kb)

Additional file 3: Expression of arsenic transport and metabolism genes does not appear to be different between male and female placenta. Boxplots comparing expression in male and female placenta of (A) *AS3MT*, (B) *GSTM1*, (C) *AQP9*, and (D) *SLC39A2*. Upper and lower ends of boxes indicate the 25th and 75th percentiles, respectively, and black band represents the median. Error bars represent minimum and maximum values, excluding outliers, which are depicted as open dots. *P* values are based on a Wilcoxon signed rank test. (PPTX 271 kb)

Additional file 4: Adjustment for *AQP9* expression attenuates the associations of a subset of developmental genes with U-As in female fetal placenta. Multivariable linear regression analyses were performed to determine the association of U-As levels with developmental/stemness gene expression in female fetal placenta, (A) without or (B) with adjustment for *AQP9* expression. All analyses were adjusted for maternal age. *P < 0.05, **P < 0.01, ***P < 0.001. Green; HH pathway-related genes, purple; NOTCH pathway-related genes, blue; WNT pathway-related genes, orange; stemness genes. (PPTX 550 kb)

Additional file 5: Candidate gene associations with *AQP9* expression in male and female placenta, with or without adjustment for U-As levels. Multivariable linear regression analyses were performed as in Fig. 1, to determine the association of placental *AQP9* expression with developmental/stemness gene expression after cohort stratification by infant sex, with or without adjustment for U-As levels. All analyses were adjusted for maternal age. *P < 0.05, **P < 0.01, ***P < 0.001. (XLSX 65 kb)

Additional file 6: Candidate gene associations with percentages of different urinary arsenic subtypes in male and female placenta. The percentages of iAs, MMA^V^, and DMA^V^ in maternal urine were computed by dividing their urinary levels by total U-As levels. Multivariable linear regression analyses were then performed to determine the association of the percentage of arsenic subtype with developmental/stemness gene expression, after cohort stratification by infant sex (females; sheet 1, males; sheet 2). Analyses were adjusted for maternal age only. *P < 0.05, **P < 0.01. (XLSX 61 kb)

## Abbreviations

EPA MCL: Environmental Protection Agency’s Maximum Contaminant Level
IQR: interquartile range
NHBCS: New Hampshire Birth Cohort Study
U-As: total maternal urinary arsenic concentration (excluding arsenobetaine)

## References

[1] Franconi F, Brunelleschi S, Steardo L, Cuomo V (2007). Gender differences in drug responses. Pharmacol Res.

[2] Waxman DJ, Holloway MG (2009). Sex differences in the expression of hepatic drug metabolizing enzymes. Mol Pharmacol.

[3] Aiken CE, Ozanne SE (2013). Sex differences in developmental programming models. Reproduction.

[4] Gabory A, Roseboom TJ, Moore T, Moore LG, Junien C (2013). Placental contribution to the origins of sexual dimorphism in health and diseases: sex chromosomes and epigenetics. Biol Sex Differ.

[5] Clifton VL (2010). Review: Sex and the human placenta: mediating differential strategies of fetal growth and survival. Placenta.

[6] Gluckman PD, Hanson MA, Beedle AS (2007). Early life events and their consequences for later disease: a life history and evolutionary perspective. Am J Hum Biol.

[7] Hughes MF, Beck BD, Chen Y, Lewis AS, Thomas DJ (2011). Arsenic exposure and toxicology: a historical perspective. Toxicol Sci.

[8] Nordstrom DK (2002). Public health. Worldwide occurrences of arsenic in ground water. Science.

[9] Bhattacharjee P, Chatterjee D, Singh KK, Giri AK (2013). Systems biology approaches to evaluate arsenic toxicity and carcinogenicity: an overview. Int J Hyg Environ Health.

[10] Sohel N, Vahter M, Ali M, Rahman M, Rahman A, Streatfield PK, Kanaroglou PS, Persson LA (2010). Spatial patterns of fetal loss and infant death in an arsenic-affected area in Bangladesh. Int J Health Geogr.

[11] Myers SL, Lobdell DT, Liu Z, Xia Y, Ren H, Li Y, Kwok RK, Mumford JL, Mendola P (2010). Maternal drinking water arsenic exposure and perinatal outcomes in inner Mongolia, China. J Epidemiol Community Health.

[12] Llanos MN, Ronco AM (2009). Fetal growth restriction is related to placental levels of cadmium, lead and arsenic but not with antioxidant activities. Reprod Toxicol.

[13] Huyck KL, Kile ML, Mahiuddin G, Quamruzzaman Q, Rahman M, Breton CV, Dobson CB, Frelich J, Hoffman E, Yousuf J (2007). Maternal arsenic exposure associated with low birth weight in Bangladesh. J Occup Environ Med.

[14] Xu L, Yokoyama K, Tian Y, Piao FY, Kitamura F, Kida H, Wang P (2011). Decrease in birth weight and gestational age by arsenic among the newborn in Shanghai, China. Nihon Koshu Eisei Zasshi.

[15] Rahman A, Vahter M, Ekstrom EC, Persson LA (2011). Arsenic exposure in pregnancy increases the risk of lower respiratory tract infection and diarrhea during infancy in Bangladesh. Environ Health Perspect.

[16] Farzan SF, Karagas MR, Chen Y (2013). In utero and early life arsenic exposure in relation to long-term health and disease. Toxicol Appl Pharmacol.

[17] Davis MA, Higgins J, Li Z, Gilbert-Diamond D, Baker ER, Das A, Karagas MR (2015). Preliminary analysis of in utero low-level arsenic exposure and fetal growth using biometric measurements extracted from fetal ultrasound reports. Environ Health.

[18] Farzan SF, Li Z, Korrick SA, Spiegelman D, Enelow R, Nadeau K, Baker E (2015). Karagas MR.

[19] Gossai A, Lesseur C, Farzan S, Marsit C, Karagas MR, Gilbert-Diamond D (2015). Association between maternal urinary arsenic species and infant cord blood leptin levels in a New Hampshire Pregnancy Cohort. Environ Res.

[20] Green BB, Karagas MR, Punshon T, Jackson BP, Robbins DJ, Houseman EA, et al. Epigenome-Wide Assessment of DNA Methylation in the Placenta and Arsenic Exposure in the New Hampshire Birth Cohort Study (USA). Environ Health Perspect. 2016; 10.1289/ehp.1510437 PMC4977055 26771251

[21] Nadeau KC, Li Z, Farzan S, Koestler D, Robbins D, Fei DL, Malipatlolla M, Maecker H, Enelow R, Korrick S, Karagas MR (2014). In utero arsenic exposure and fetal immune repertoire in a US pregnancy cohort. Clin Immunol.

[22] Rahman A, Vahter M, Smith AH, Nermell B, Yunus M, El Arifeen S, Persson LA, Ekstrom EC (2009). Arsenic exposure during pregnancy and size at birth: a prospective cohort study in Bangladesh. Am J Epidemiol.

[23] Hopenhayn C, Ferreccio C, Browning SR, Huang B, Peralta C, Gibb H, Hertz-Picciotto I (2003). Arsenic exposure from drinking water and birth weight. Epidemiology.

[24] Hamadani JD, Tofail F, Nermell B, Gardner R, Shiraji S, Bottai M, Arifeen SE, Huda SN, Vahter M (2011). Critical windows of exposure for arsenic-associated impairment of cognitive function in pre-school girls and boys: a population-based cohort study. Int J Epidemiol.

[25] Winterbottom EF, Fei DL, Koestler DC, Giambelli C, Wika E, Capobianco AJ, Lee E, Marsit CJ, Karagas MR, Robbins DJ (2015). GLI3 links environmental arsenic exposure and human fetal growth. EBioMedicine.

[26] Fei DL, Koestler DC, Li Z, Giambelli C, Sanchez-Mejias A, Gosse JA, Marsit CJ, Karagas MR, Robbins DJ (2013). Association between In Utero arsenic exposure, placental gene expression, and infant birth weight: a US birth cohort study. Environ Health.

[27] Gilbert-Diamond D, Cottingham KL, Gruber JF, Punshon T, Sayarath V, Gandolfi AJ, Baker ER, Jackson BP, Folt CL, Karagas MR (2011). Rice consumption contributes to arsenic exposure in US women. Proc Natl Acad Sci U S A.

[28] Koestler DC, Avissar-Whiting M, Houseman EA, Karagas MR, Marsit CJ (2013). Differential DNA methylation in umbilical cord blood of infants exposed to low levels of arsenic in utero. Environ Health Perspect.

[29] Johnson WE, Li C, Rabinovic A (2007). Adjusting batch effects in microarray expression data using empirical Bayes methods. Biostatistics.

[30] Liu Z, Styblo M, Rosen BP (2006). Methylarsonous acid transport by aquaglyceroporins. Environ Health Perspect.

[31] Mukhopadhyay R, Bhattacharjee H, Rosen BP (1840). Aquaglyceroporins: generalized metalloid channels. Biochim Biophys Acta.

[32] Damiano AE (2011). Review: Water channel proteins in the human placenta and fetal membranes. Placenta.

[33] Khairul I, Wang QQ, Jiang YH, Wang C, Naranmandura H. Metabolism, toxicity and anticancer activities of arsenic compounds. Oncotarget. 2017;8:23905–26. 10.18632/oncotarget.14733 PMC5410354 28108741

[34] Torres-Avila M, Leal-Galicia P, Sanchez-Pena LC, Del Razo LM, Gonsebatt ME (2010). Arsenite induces aquaglyceroporin 9 expression in murine livers. Environ Res.

[35] Lebeck J, Gena P, O'Neill H, Skowronski MT, Lund S, Calamita G, Praetorius J (2012). Estrogen prevents increased hepatic aquaporin-9 expression and glycerol uptake during starvation. Am J Physiol Gastrointest Liver Physiol.

[36] Lebeck J (2014). Metabolic impact of the glycerol channels AQP7 and AQP9 in adipose tissue and liver. J Mol Endocrinol.

[37] Druwe IL, Vaillancourt RR (2010). Influence of arsenate and arsenite on signal transduction pathways: an update. Arch Toxicol.

[38] Maciaszczyk-Dziubinska E, Wawrzycka D, Wysocki R (2012). Arsenic and antimony transporters in eukaryotes. Int J Mol Sci.

[39] Hall M, Gamble M, Slavkovich V, Liu X, Levy D, Cheng Z, van Geen A, Yunus M, Rahman M, Pilsner JR, Graziano J (2007). Determinants of arsenic metabolism: blood arsenic metabolites, plasma folate, cobalamin, and homocysteine concentrations in maternal-newborn pairs. Environ Health Perspect.

[40] Punshon T, Davis MA, Marsit CJ, Theiler SK, Baker ER, Jackson BP, Conway DC, Karagas MR (2015). Placental arsenic concentrations in relation to both maternal and infant biomarkers of exposure in a US cohort. J Expo Sci Environ Epidemiol.

[41] Caldwell KL, Jones RL, Verdon CP, Jarrett JM, Caudill SP, Osterloh JD (2009). Levels of urinary total and speciated arsenic in the US population: National Health and Nutrition Examination Survey 2003-2004. J Expo Sci Environ Epidemiol.

[42] Damiano AE, Zotta E, Ibarra C (2006). Functional and molecular expression of AQP9 channel and UT-A transporter in normal and preeclamptic human placentas. Placenta.

[43] Zhang HJ, Siu MK, Wong ES, Wong KY, Li AS, Chan KY, Ngan HY, Cheung AN (2008). Oct4 is epigenetically regulated by methylation in normal placenta and gestational trophoblastic disease. Placenta.

[44] Concha G, Vogler G, Lezcano D, Nermell B, Vahter M (1998). Exposure to inorganic arsenic metabolites during early human development. Toxicol Sci.

[45] Al-Qattan MM, Shamseldin HE, Salih MA, Alkuraya FS. GLI3-related polydactyly: a review. Clin Genet. 2017; doi:10.1111/cge.12952. 10.1111/cge.12952 28224613

[46] Grzeschik KH, Bornholdt D, Oeffner F, Konig A, del Carmen BM, Enders H, Fritz B, Hertl M, Grasshoff U, Hofling K (2007). Deficiency of PORCN, a regulator of Wnt signaling, is associated with focal dermal hypoplasia. Nat Genet.

[47] Wang X, Reid Sutton V, Omar Peraza-Llanes J, Yu Z, Rosetta R, Kou YC, Eble TN, Patel A, Thaller C, Fang P, Van den Veyver IB (2007). Mutations in X-linked PORCN, a putative regulator of Wnt signaling, cause focal dermal hypoplasia. Nat Genet.

[48] Rosenfeld CS (2015). Sex-Specific Placental Responses in Fetal Development. Endocrinology.

