# Supplementary figures and images for: The aquaglyceroporin AQP9 contributes to the sex-specific effects of in utero arsenic exposure on placental gene expression
Source: Environ Health. 2017 Jun 14;16:59. doi: 10.1186/s12940-017-0267-8 (PMC5471920; doi:10.1186/s12940-017-0267-8)

## Slide 1
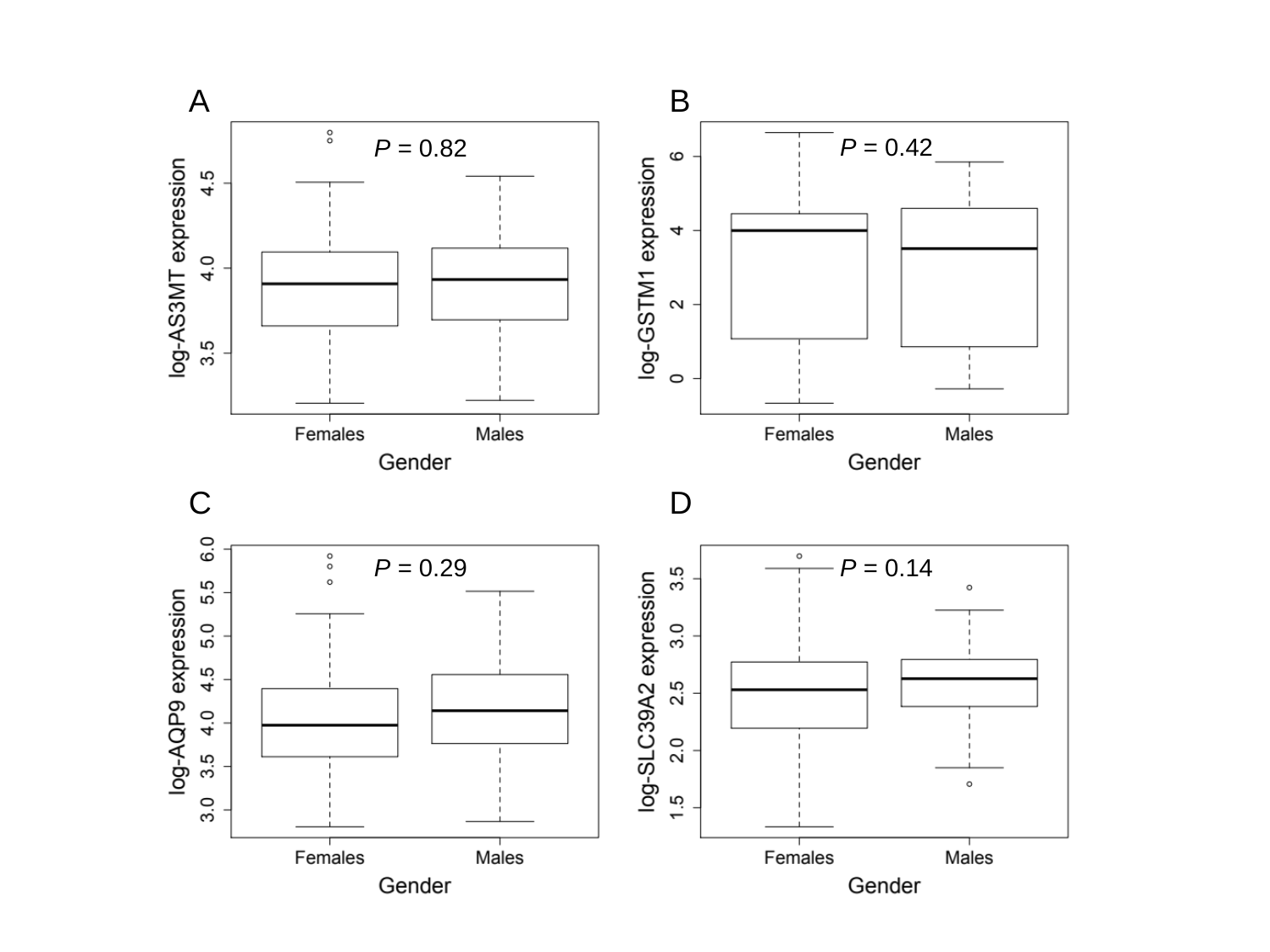

A
B
P = 0.42
P = 0.82
C
D
P = 0.29
P = 0.14

Supplement: Supplementary file 3 — Expression of arsenic transport and metabolism genes does not appear to be different between male and female placenta. Boxplots comparing expression in male and female placenta of (A) AS3MT, (B) GSTM1, (C) AQP9, and (D) SLC39A2. Upper and lower ends of boxes indicate the 25th and 75th percentiles, respectively, and black band represents the median. Error bars represent minimum and maximum values, excluding outliers, which are depicted as open dots. P values are based on a Wilcoxon signed rank test. (PPTX 271 kb) [file 12940_2017_267_MOESM3_ESM.pptx]
