# Supplementary material for: The aquaglyceroporin AQP9 contributes to the sex-specific effects of in utero arsenic exposure on placental gene expression
Source: Environ Health. 2017 Jun 14;16:59. doi: 10.1186/s12940-017-0267-8 (PMC5471920; doi:10.1186/s12940-017-0267-8)
Supplement: Supplementary file 4 — Adjustment for AQP9 expression attenuates the associations of a subset of developmental genes with U-As in female fetal placenta. Multivariable linear regression analyses were performed to determine the association of U-As levels with developmental/stemness gene expression in female fetal placenta, (A) without or (B) with adjustment for AQP9 expression. All analyses were adjusted for maternal age. *P < 0.05, **P < 0.01, ***P < 0.001. Green; HH pathway-related genes, purple; NOTCH pathway-related genes, blue; WNT pathway-related genes, orange; stemness genes. (PPTX 550 kb) [file 12940_2017_267_MOESM4_ESM.pptx]

## Slide 1
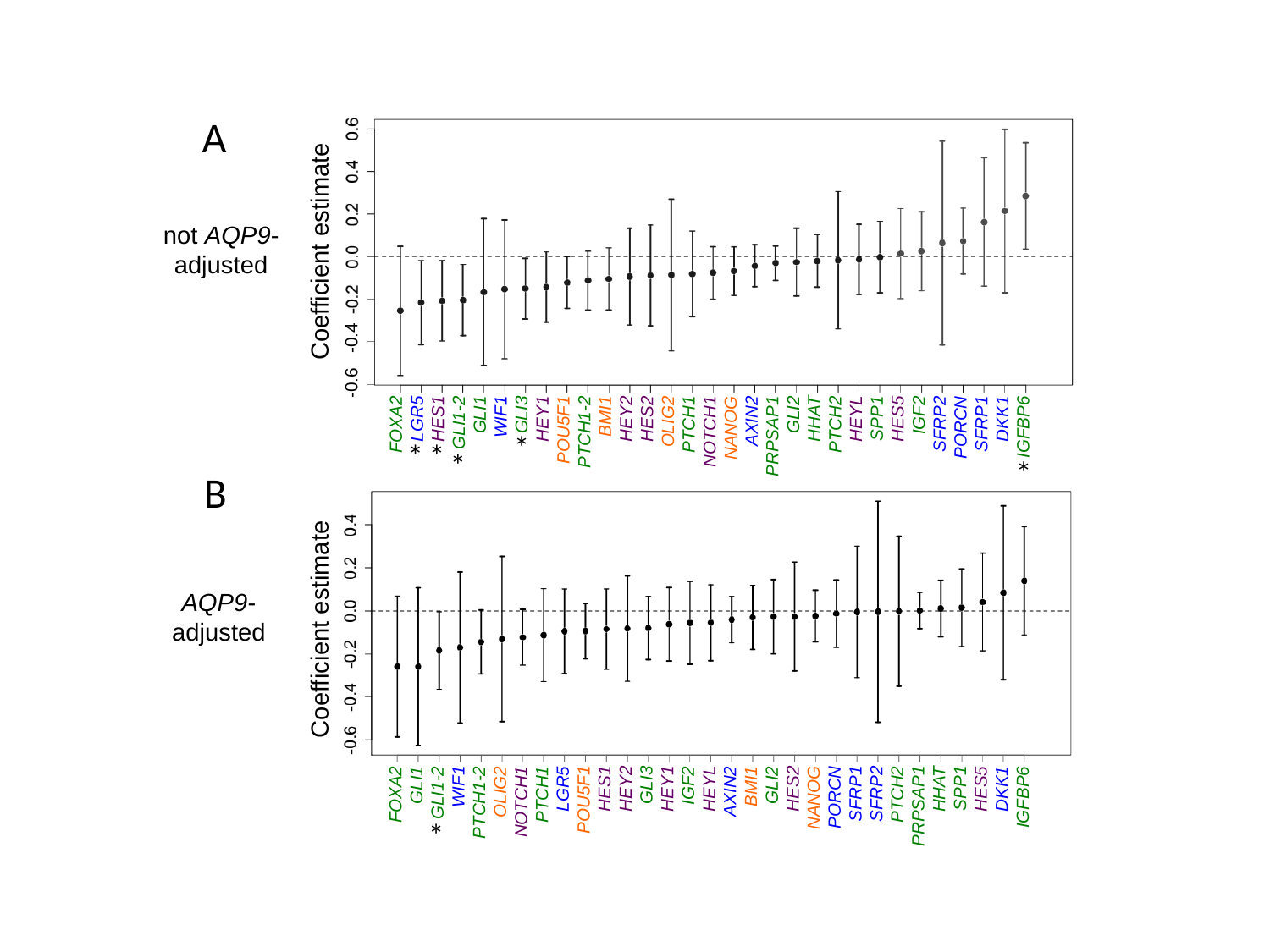

FOXA2
LGR5
HES1
GLI1-2
GLI1
WIF1
GLI3
HEY1
POU5F1
PTCH1-2
BMI1
HEY2
HES2
OLIG2
PTCH1
NOTCH1
NANOG
AXIN2
PRPSAP1
GLI2
HHAT
PTCH2
HEYL
SPP1
HES5
IGF2
SFRP2
PORCN
SFRP1
DKK1
IGFBP6
Coefficient estimate
-0.6 -0.4
*
*
*
*
*
A
not AQP9-adjusted
B
FOXA2
GLI1
GLI1-2
WIF1
PTCH1-2
OLIG2
NOTCH1
PTCH1
LGR5
POU5F1
HES1
HEY2
GLI3
HEY1
IGF2
HEYL
AXIN2
BMI1
GLI2
HES2
NANOG
PORCN
SFRP1
SFRP2
PTCH2
PRPSAP1
HHAT
SPP1
HES5
DKK1
IGFBP6
Coefficient estimate
*
AQP9-adjusted
